# Multiple adhesin proteins on the cell surface of *Streptococcus gordonii* are involved in adhesion to human fibronectin

**DOI:** 10.1099/mic.0.032078-0

**Published:** 2009-11

**Authors:** Nicholas S. Jakubovics, Jane L. Brittan, Lindsay C. Dutton, Howard F. Jenkinson

**Affiliations:** 1 School of Dental Sciences, Newcastle University, Framlington Place, Newcastle upon Tyne NE2 4BW, UK; 2 Department of Oral and Dental Sciences, University of Bristol, Lower Maudlin Street, Bristol BS1 2LY, UK

## Abstract

Adhesion of bacterial cells to fibronectin (FN) is thought to be a pivotal step in the pathogenesis of invasive infectious diseases. Viridans group streptococci such as *Streptococcus gordonii* are considered commensal members of the oral microflora, but are important pathogens in infective endocarditis. *S. gordonii* expresses a battery of cell-surface adhesins that act alone or in concert to bind host receptors. Here, we employed molecular genetic approaches to determine the relative contributions of five known *S. gordonii* surface proteins to adherence to human FN. Binding levels to FN by isogenic mutants lacking Hsa glycoprotein were reduced by 70 %, while mutants lacking CshA and CshB fibrillar proteins showed approximately 30 % reduced binding. By contrast, disruption of antigen I/II adhesin genes s*spA* and *sspB* in a wild-type background did not result in reduced FN binding. Enzymic removal of sialic acids from FN led to reduced *S. gordonii* DL1 adhesion (>50 %), but did not affect binding by the *hsa* mutant, indicating that Hsa interacts with sialic acid moieties on FN. Conversely, desialylation of FN did not affect adherence levels of *Lactococcus lactis* cells expressing SspA or SspB polypeptides. Complementation of the *hsa* mutant partially restored adhesion to FN. A model is proposed for FN binding by *S. gordonii* in which Hsa and CshA/CshB are primary adhesins, and SspA or SspB play secondary roles. Understanding the basis of oral streptococcal interactions with FN will provide a foundation for development of new strategies to control infective endocarditis.

## INTRODUCTION

Adhesive interactions between bacterial cell-surface molecules and host proteins or glycoproteins form the first step in colonization by commensal microbes and pathogens alike. In humans, fibronectin (FN) is a major target of bacterial adhesion mechanisms. FN is a high-molecular-mass glycoprotein that is present in adults in two forms: soluble FN circulates in the bloodstream and cellular FN is found in the extracellular matrix. Adhesion of bacteria to cellular FN is thought to be a central element in a variety of infectious diseases (Joh *et al.*, 1999). The molecular interactions underlying bacterial binding to FN have been particularly well studied in the Gram-positive pathogens *Staphylococcus aureus* and *Streptococcus pyogenes* (Schwarz-Linek *et al.*, 2006). These interactions involve recognition of the FN peptide backbone by bacterial adhesins through a tandem *β*-zipper motif (Schwarz-Linek *et al.*, 2003).

Bacterial infective endocarditis is a rare, but extremely dangerous, infection of the cardiac endothelium usually at the site of the heart valves. Infective endocarditis most commonly affects patients with pre-existing defects or damage to the heart valve tissue, or with prosthetic heart valves. Even with available antibiotic therapies, mortality from infective endocarditis remains at levels approaching 25 % (Sy *et al.*, 2009). Gram-positive cocci account for >80 % of bacterial infective endocarditis cases, and oral viridans streptococci, including *Streptococcus gordonii*, *Streptococcus sanguinis* and *Streptococcus mitis*, are the most common causative agents of native valve endocarditis (Tomás Carmona *et al.*, 2007). A key step in the pathogenesis of infective endocarditis is the attachment of bacteria to the endocardial lining. Oral streptococci possess a variety of adhesins that recognize host cells and extracellular components, including FN.

The major cell-surface adhesins of *S. gordonii* include antigen I/II (AgI/II) family proteins SspA and SspB (Jakubovics *et al.*, 2005b), cell-surface fibrillar proteins CshA and CshB (McNab *et al.*, 1999), sialic-acid-binding protein Hsa/GspB (Bensing & Sullam, 2002; Takahashi *et al.*, 2002) and amylase-binding proteins AbpA and AbpB (Tanzer *et al.*, 2003). Of these, only Hsa and its allelic variant GspB have been directly examined for their roles in endocarditis. Disruption of *hsa* in *S. gordonii* DL1 (Challis) or *gspB* in *S. gordonii* M99 significantly reduce virulence in the rat model of endocarditis (Takahashi *et al.*, 2006; Xiong *et al.*, 2008). Thus, Hsa/GspB is an important virulence determinant in the pathogenesis of streptococcal endocarditis.

The identification of streptococcal adhesins involved in interactions with specific host substrates is often complicated by the presence of multiple redundant adhesins on the streptococcal cell surface. Thus, binding of *S. gordonii* to salivary agglutinin glycoprotein gp340, an innate defence molecule, involves the concerted activities of SspA, SspB and Hsa (Jakubovics *et al.*, 2005a). Purified SspA and SspB proteins interact with FN and with *β*1 integrins (Nobbs *et al.*, 2007). Adhesion of *S. gordonii* cells to FN also appears to involve a number of streptococcal proteins. Adhesins CshA and CshB are required for maximum binding to FN (McNab *et al.*, 1996). However, a double mutant disrupted in genes encoding both CshA and CshB retained approximately 70 % of wild-type levels of FN binding (McNab *et al.*, 1996), indicating that additional adhesins contributed to FN recognition by *S. gordonii*. *S. gordonii* FbpA, a homologue of *S. pyogenes* FN-binding protein FBP54, is involved in FN binding primarily through regulation of CshA/CshB expression (Christie *et al.*, 2002). In addition, mutants in the gene encoding peptide methionine sulfoxide reductase, MsrA, are significantly impaired in adhesion to FN (Giomarelli *et al.*, 2006). MsrA is required for the stabilization of bacterial cell-surface adhesins (Wizemann *et al.*, 1996), and therefore loss of this protein probably influences multiple adhesin–receptor interactions. In this study, we aimed to characterize the roles of major adhesins CshA, CshB, SspA, SspB and Hsa in FN adhesion by *S. gordonii*. Isogenic mutants lacking individual adhesins or combinations of adhesins were constructed and tested for their capacities to bind FN. Furthermore, Hsa and AgI/II adhesins SspA and SspB were expressed in adhesion-deficient strains of *S. gordonii* or *Lactococcus lactis*. The results indicate that CshA/CshB and Hsa are major *S. gordonii* adhesins for FN, and that AgI/II adhesins SspA and SspB have minor roles. A model to describe the multifaceted nature of FN binding by *S. gordonii* is presented.

## METHODS

### Bacterial strains and growth conditions.

*S. gordonii* DL1 (Challis) and isogenic mutants UB645 (*cshA31* : : *cat cshB2* : : *ermAM*; identical to OB277), UB1360 (*sspAB* : : *aad9*), UB1545 (*hsa* : : *aphA3*) and UB1552 (*sspAB* : : *aad9 hsa* : : *aphA3*) have been described previously (Heddle *et al.*, 2003; Jakubovics *et al.*, 2005a; McNab *et al.*, 1994). *S. gordonii* strains were routinely cultured at 37 °C without shaking in BHY medium containing (l^−1^) 37 g Brain Heart Infusion (Difco) and 5 g yeast extract. Alternatively, cells were incubated at 37 °C on solidified medium (BHYN) composed of BHY supplemented with (l^−1^) 5 g Neo-peptone (Difco) and 15 g Bacto-agar. For adhesion assays, bacteria were cultured at 37 °C in TYG medium containing (l^−1^) 10 g Bacto-tryptone (Difco), 5 g yeast extract, 3 g K_2_HPO_4_ and 2 g d-glucose, and adjusted to pH 7.5 prior to autoclaving. *L. lactis* MG1363, and plasmid-bearing derivative strains (Jakubovics *et al.*, 2005b) were grown without shaking at 30 °C in M17 medium containing 5 g d-glucose l^−1^ (GM17) (Jakubovics *et al.*, 2005b). For maintenance of chromosomal deletion constructs, antibiotics were included at the following concentrations (μg ml^−1^): chloramphenicol, 5; erythromycin, 2; spectinomycin, 250; kanamycin, 250. Plasmids based on pMSP7517 or pUB1000 were maintained by inclusion of 5 μg erythromycin ml^−1^ in the growth medium.

### Genetic manipulations.

Routine cloning procedures were carried out in accordance with protocols described by Sambrook *et al.* (1989). Chromosomal DNA was extracted from streptococci following cell lysis with mutanolysin (Jenkinson, 1987). Transformation of streptococci was performed as described by Haisman & Jenkinson (1991). *S. gordonii* UB1927 (*cshA31* : : *cat cshB2* : : *ermAM sspAB* : : *aad9*) was generated by transforming *S. gordonii* UB645 with chromosomal DNA extracted from *S. gordonii* UB1360 and selecting for chloramphenicol, erythromycin and spectinomycin resistance. Similarly, *S. gordonii* UB1928 (*cshA31* : : *cat cshB2* : : *ermAM hsa* : : *aphA3*) was constructed by introducing chromosomal DNA from *S. gordonii* UB1545 into *S. gordonii* UB645 by natural transformation and selecting for chloramphenicol, erythromycin and kanamycin resistance. Transformation of *S. gordonii* UB1927 with DNA extracted from *S. gordonii* UB1545, and selection for resistance to four antibiotics (chloramphenicol, erythromycin, spectinomycin and kanamycin) generated *S. gordonii* UB1929 (*cshA31* : : *cat cshB2* : : *ermAM sspAB* : : *aad9 hsa* : : *aphA3*).

### Determination of adhesion to FN and fetuin.

Human plasma FN (Roche Diagnostics) and bovine fetuin (Sigma-Aldrich) were employed as substrates for adhesion assays. These substrates were diluted in coating buffer (20 mM Na_2_CO_3_, 20 mM NaCO_3_, pH 9.3) and immobilized on the surfaces of Immobilon microtitre plate wells (Nunc) at 4 °C for 20 h. Wells were washed with PBS, and non-specific binding sites were blocked with 1 % (w/v) BSA for 1 h at 20 °C. Wells were washed with TBSC (10 mM Tris/HCl, 150 mM NaCl, 5 mM CaCl_2_, pH 7.6). For some experiments, immobilized FN or fetuin was treated with 0.001 units sialidase (*Clostridium perfringens* neuramindase type X from Sigma-Aldrich) in reaction buffer (0.1 M sodium acetate buffer, 2 mM CaCl_2_, pH 5.0) at 37 °C for 2 h prior to addition of bacterial cells. Streptococci were cultured at 37 °C for 20 h in TYG medium, supplemented where appropriate with 100 ng nisin ml^−1^. Lactococci were incubated for 20 h in GM17 medium. Bacterial cells were harvested, washed in TBSC, and adjusted to OD_600_=1.0 in TBSC (approx. 10^9^ cells ml^−1^) using an ATI Unicam UV2 spectrophotometer. Cell suspensions (0.1 ml) were added to microtitre plate wells and incubated at 37 °C for 2 h. Wells were washed three times with TBSC, and 0.1 ml 25 % (v/v) formaldehyde was added to fix bound cells. Plates were incubated at 20 °C for 15 min, washed three times with PBS and cells were stained by the addition of 0.5 % (w/v) crystal violet (0.1 ml) and incubation for 1 min at 20 °C. Wells were washed three times with PBS. Residual dye was dissolved in 50 μl 7 % (v/v) acetic acid and quantified by measuring *A*_595_. Numbers of cells adhered were calculated from standard curves relating *A*_595_ readings to numbers of bacterial cells in microtitre plate wells. Statistical analyses were performed using Student's *t* test.

### Heterologous expression of the *S. gordonii hsa* gene.

For expression of the *S. gordonii hsa* gene under control of the nisin induction system, plasmid pMSP7517 (Hirt *et al.*, 2000) was modified as follows. pMSP7517 contains the *Enterococcus faecalis prgB* gene, encoding aggregation substance, downstream of the nisin-inducible promoter P*_nisA_*. Also included in pMSP7517 are *nisK* and *nisR*, encoding the nisin sensor and regulator, respectively, that are required for nisin induction, and the *ermAM* erythromycin resistance cassette. The *hsa* gene, and associated ribosome-binding site and transcription terminator element, was amplified from *S. gordonii* DL1 (Challis) using the proofreading polymerase Platinum *pfx* (Invitrogen) and primers *hsaPf4* (5′-TGCTCCCATGGAAATTAAGTAGAGGGGATTACATG-3′; *Nco*I site underlined) and *hsaPr4* (5′-TGCACCTCGAGAAAAGCTAGAACATCAAGGACT-3′; *Xho*I site underlined). The PCR product (6655 bp) was digested with *Nco*I and *Xho*I. The *prgB* gene was excised from plasmid pMSP7517 by digestion with *Nco*I and *Xho*I. The vector was ligated to the *Nco*I/*Xho*I-digested *hsa* PCR amplification product, and the resultant plasmid, pMSP-*hsa*, was transformed into *E. coli* DH5*α*. The presence of the *hsa* gene in the correct orientation in pMSP-*hsa* was confirmed by restriction enzyme digestion analysis and by DNA sequencing. Plasmid pMSP-*hsa* was extracted from *E. coli* and transformed into *S. gordonii hsa* mutants UB1545 and UB1552.

### Lectin blotting.

To monitor the induction of the *hsa* gene product in *S. gordonii* UB1545(pMSP-*hsa*), cells were cultured at 37 °C for 20 h in TYG medium. Cultures were diluted 100-fold in fresh TYG. At the early exponential phase of growth (OD_600_=0.3), nisin was added to a concentration of 100 ng ml^−1^. *S. gordonii* cell-wall proteins were extracted by incubation with mutanolysin (Jakubovics *et al.*, 2000), and 20 μg portions were separated under denaturing conditions on 5 % polyacrylamide gels. Proteins were transferred to Amersham Hybond ECL nitrocellulose membranes (GE Healthcare) at 100 V for 1.5 h. Membranes were probed with biotin-labelled succinylated wheatgerm agglutinin (sWGA), and detected with 0.2 μg peroxidase-conjugated streptavidin ml^−1^. Densitometric analysis of band intensities was performed using ImageJ software (Collins, 2007).

## RESULTS

### Adhesion of *cshA*/*B*, *sspA*/*B* and *hsa* mutants to FN

**Fig. 1. f1:**
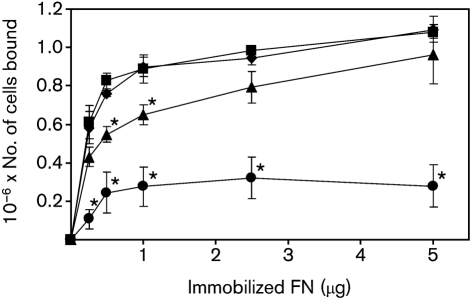
FN binding by *S. gordonii* DL1 (⧫) and isogenic mutants UB1360 Δ(*sspA sspB*) (▪), UB645 Δ(*cshA cshB*) (▴), and UB1545 Δ*hsa* (•). FN was immobilized on the surface of microtitre wells and incubated in the presence of bacteria. After washing, bound bacterial cells were quantified by staining with crystal violet (see Methods). Means±sem from three independent experiments are shown. Values that were significantly lower (*P*<0.05) than adhesion levels of *S. gordonii* DL1 at the equivalent FN concentration are marked with an asterisk above and to the right of the data point.

FN-binding mechanisms have been well-described in the Gram-positive organisms *Sta. aureus* and *S. pyogenes* (Schwarz-Linek *et al.*, 2006), and involve recognition of the FN peptide backbone by bacterial cell-surface adhesins. In *S. pyogenes*, several different cell-surface proteins contribute to the overall interaction with FN. We hypothesized that binding of *S. gordonii* cells to FN may be mediated by a combination of cell-surface adhesins. To test this, FN binding by isogenic mutants lacking specific cell-surface adhesins, or adhesin protein families, was assessed using a microtitre plate adhesion assay (see Methods). In agreement with a previous study (McNab *et al.*, 1996), adhesion of wild-type *S. gordonii* DL1 increased with increasing FN over the range 0.25–5 μg FN per well (Fig. 1). Between 1 and 5 μg FN per well, there was only a small (<20 %) increase in the number of cells bound. Adhesion of isogenic mutants lacking *cshA* and *cshB* genes to FN was up to 28 % lower than wild-type at all FN concentrations tested. Adhesion of the *cshA*/*cshB* mutant to 0.5 or 1.0 μg FN was significantly reduced (*P*<0.05) compared with wild-type. At higher concentrations of FN (2.5 or 5 μg), the reduction in binding of the *cshA*/*cshB* mutant compared with wild-type was not statistically significant (*P*>0.05) (Fig. 1; McNab *et al.*, 1996). Binding of the *hsa* mutant to FN was reduced by between 66 and 82 % compared with wild-type at all concentrations tested, and these differences were all statistically significant (*P*<0.05). By contrast, disruption of AgI/II genes *sspA*/*sspB* did not result in reduced FN binding levels compared with the isogenic wild-type progenitor (Fig. 1).

### Effects of combined cell-surface adhesin gene disruptions on adhesion to FN and fetuin

**Fig. 2. f2:**
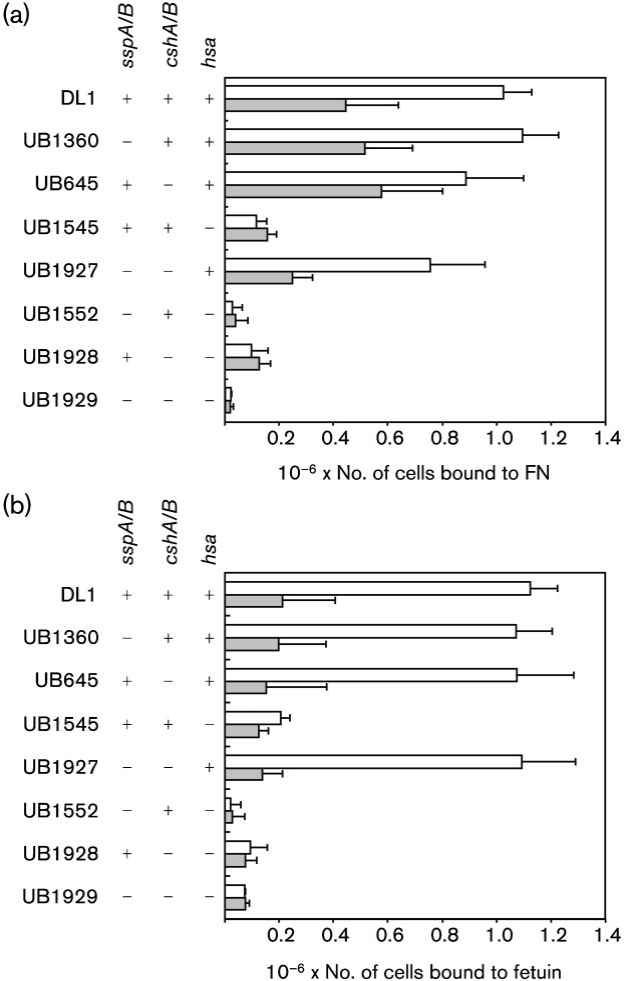
FN (a) and fetuin (b) adhesion by *S. gordonii* DL1 and isogenic mutants lacking different combinations of cell-surface adhesins. Binding was measured to untreated (white bars) or sialidase-treated (shaded bars) substrate. Bars represent mean values±sem from four independent experiments.

To assess whether cell-surface adhesins of *S. gordonii* are functionally redundant for FN binding, isogenic mutants were constructed in which multiple proteins were disrupted. All combinations of mutants lacking functional *cshA*/*cshB*, *sspA*/*sspB* and *hsa* genes were produced, and adhesion of wild-type *S. gordonii* DL1 and mutant strains to FN (2.5 μg) was assessed (Fig. 2a[Fig f2]). At this FN concentration there was reduced binding of the *cshA/cshB* mutant to FN, but this was not statistically significant. Similarly, in strains mutated in *sspA*/*sspB* or in *hsa* genes, disruption of *cshA*/*cshB* resulted in reduced binding to FN compared with strains bearing intact *cshA* and *cshB* genes. However, these reductions did not reach statistical significance (Fig. 2a[Fig f2], for example compare UB1927 with UB1360). It seems, therefore, that significantly reduced FN (1 μg) binding resulting from disruption of *cshA* and *cshB* (shown in Fig. 1) was not so evident with higher FN concentrations utilized for the results shown in Fig. 2(a).

Disruption of *hsa* significantly (*P*<0.05) reduced adhesion of *S. gordonii* to FN in the presence or absence of intact *sspA*/*sspB* or *cshA*/*cshB* genes (Fig. 2a[Fig f2]). When either *cshA*/*cshB* or *hsa* genes were knocked out, reduced levels of adhesion were observed for strains mutated in AgI/II protein genes *sspA*/*sspB* compared with strains possessing functional *sspA*/*sspB* (Fig. 2a[Fig f2]; compare UB1927 with UB645, UB1929 with UB1928, and UB1552 with UB1545). Although none of these differences were statistically significant, they were consistent and indicated that AgI/II proteins might have secondary roles in influencing adhesion to FN.

### Hsa protein interacts with FN

FN is a glycoprotein containing approximately 1.2 % sialic acids (Carsons *et al.*, 1987). Substrate interactions by *S. gordonii* Hsa are dependent upon recognition of sialic acids (Takamatsu *et al.*, 2005). We reasoned, therefore, that recognition of FN by *S. gordonii* may involve sialic acid moieties on the termini of FN carbohydrate chains. To test this, FN was treated with sialidase (neuraminidase) prior to the addition of bacterial cells. Adhesion of wild-type *S. gordonii* DL1 was reduced >50 % following sialidase treatment of FN (Fig. 2a[Fig f2]). Similarly, FN binding by *sspA*/*sspB* and *cshA*/*cshB* mutants containing intact *hsa* (strains UB1360, UB645 and UB1927) was reduced 30–60 % by sialidase treatment. Adhesion of *hsa* mutant strains UB1545, UB1552, UB1928 and UB1929 to FN was low and was not further reduced by incubation of FN with sialidase (Fig. 2a[Fig f2]).

The above data indicated that Hsa is a major adhesin for FN. We have previously demonstrated that Hsa protein levels are not affected by disruption of AgI/II protein genes *sspA*/*sspB* (Jakubovics *et al.*, 2005a). To ensure that disruption of *cshA*/*cshB* did not modulate expression of Hsa, cell-surface proteins were extracted from *S. gordonii* DL1 and *S. gordonii* UB645. Serial twofold dilutions were applied to nitrocellulose using a dot blotter, and the membrane was probed with sWGA, which specifically recognizes Hsa glycoprotein in *S. gordonii* cell-wall extracts (Jakubovics *et al.*, 2005a). Levels of Hsa expression were not changed by disruption of *cshA*/*cshB* in strain UB645 (data not shown). To further test that Hsa function was not affected by deletion of cell-wall protein genes *cshA, cshB, sspA* and *sspB*, adhesion of *S. gordonii* cell-surface adhesin mutants to bovine fetuin was assessed. Fetuin is a heavily glycosylated protein that has been used as a model substrate for Hsa binding (Takamatsu *et al.*, 2005). *S. gordonii* DL1 adhered to fetuin in a dose-dependent manner (Fig. 2b[Fig f2] and data not shown). Pre-treatment of fetuin with sialidase reduced binding by >80 %. Levels of fetuin binding by mutant *S. gordonii* strains UB1360, UB645 and UB1927, lacking SspA/SspB and/or CshA/CshB, were almost identical to wild-type binding levels (Fig. 2b[Fig f2]). In each case, adhesion of these mutants was reduced >80 % following sialidase treatment of the fetuin. In contrast, binding of *S. gordonii* UB1545 *hsa* to fetuin was extremely low (∼15 % of wild-type binding), and was not further reduced by desialylation of the fetuin substrate. Fetuin binding by isogenic mutants lacking Hsa in addition to SspA/SspB and/or CshA/CshB (strains UB1552, UB1928 and UB1929) was barely detectable. These data indicate that Hsa function, in terms of adhesion to fetuin, was not impaired by disruption of SspA/SspB and/or CshA/CshB.

### Complementation of the *hsa* knockout using a nisin-inducible promoter construct

**Fig. 3. f3:**
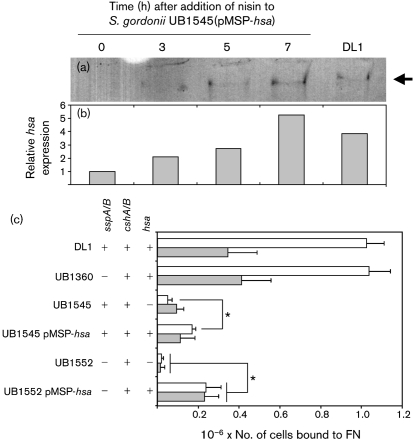
Effect of Hsa complementation on adhesion of *S. gordonii* to FN. The expression of Hsa from pMSP-*hsa* was monitored by lectin blotting (a). Cell-wall proteins were extracted from *S. gordonii* UB1545(pMSP-*hsa*) at different times following induction of *hsa* expression by addition of nisin to the culture. Equal amounts (20 μg) of proteins were separated by PAGE and blotted onto nitrocellulose. Membranes were probed with sWGA, which specifically recognizes Hsa, and a single band was detected at >250 kDa in each lane (arrow). Extracts from *S. gordonii* DL1 were included for comparison. The amount of sWGA-reactive protein in each lane was quantified by densitometry (b). (c) Adhesion of *S. gordonii* strains to immobilized FN was determined by staining with crystal violet, and mean values±sem from three independent experiments are shown. Significant differences between adhesion of UB1545 and UB1545(pMSP-*hsa*) to untreated FN, and between UB1552 and UB1552(pMSP-*hsa*) to untreated (white bars) or sialidase-treated (shaded bars) FN (Student's *t*-test, *P*<0.05), are indicated by asterisks.

To examine further the role of Hsa in adhesion to FN, *hsa* was cloned downstream of a nisin-inducible promoter (Bryan *et al.*, 2000) to create plasmid pMSP-*hsa*, and introduced into *S. gordonii* UB1545 *hsa* and *S. gordonii* UB1552 *hsa sspAB*. Expression of Hsa in *S. gordonii* UB1545(pMSP-*hsa*) was monitored following addition of nisin by lectin blotting using biotinylated sWGA. The Hsa glycoprotein is difficult to extract and forms a diffuse band on SDS-PAGE (Takahashi *et al.*, 2002; Takahashi *et al.*, 2004). An sWGA-positive band (>250 kDa) was detected in cell-wall extracts of *S. gordonii* DL1, and this was barely visible in the complemented mutant prior to nisin addition (Fig. 3a[Fig f3]). Within 3 h after addition of nisin to *S. gordonii* UB1545(pMSP-*hsa*) culture, Hsa expression was greater than twofold higher than the initial level. Hsa expression continued to increase for 7 h after induction (Fig. 3a, b[Fig f3]). Adhesion to FN was partially restored by expression of *hsa* from plasmid pMSP-*hsa* in *S. gordonii* UB1545 (Fig. 3c[Fig f3]). In contrast, there was no difference in FN adhesion between *S. gordonii* UB1545 and *S. gordonii* UB1545(pMSP7517), expressing the ‘empty’ vector (data not shown). Binding levels of *S. gordonii* UB1552(pMSP-*hsa*) to sialidase-treated FN and untreated FN were similar, and were both significantly greater than the *hsa*/*sspA*/*sspB* mutant *S. gordonii* UB1552 (Fig. 3c[Fig f3]). These data indicate that expression of Hsa increases binding to FN, and also to asialo-FN in the absence of SspA and SspB. The increase in binding of *S. gordonii* UB1552 to asialo-FN upon complementation with plasmid pMSP-*hsa* was unexpected, since Hsa-mediated adhesion is thought to be highly specific for sialic acid residues (Takamatsu *et al.*, 2005). Prolonged incubation in the presence of the inducer nisin led to slightly higher levels of Hsa in strains bearing plasmid pMSP-*hsa* than in strains with a chromosomal *hsa* gene (Fig. 3a and b[Fig f3]). It is possible that in the absence of SspA or SspB, increased levels of Hsa promote adhesion of *S. gordonii* to non-sialic acid carbohydrates on FN or to the FN backbone. Alternatively, the altered expression levels of cell-surface adhesins in this strain may have led to gross changes in cell-wall structure. By comparison, expression of Hsa from plasmid pMSP-*hsa* in *S. gordonii* UB1545 or in *S. gordonii* UB1552 partially restored binding to fetuin, but not to asialofetuin (data not shown).

### Heterologous expression of SspA/SspB in *L. lactis* increases adhesion to FN

**Fig. 4. f4:**
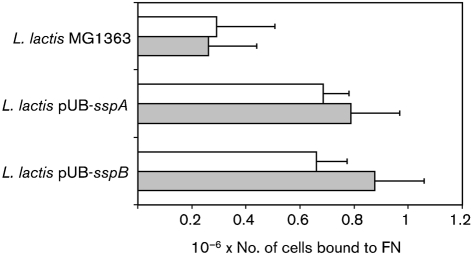
FN adhesion by *L. lactis* MG1363 and derivative strains expressing AgI/II polypeptides SspA or SspB from *S. gordonii*. Binding to untreated (white bars) or sialidase-treated (shaded bars) FN was measured. Mean values±sem from four independent experiments are shown.

The results presented above indicated that AgI/II proteins of *S. gordonii* may have more minor roles in adhesion to FN compared with the adhesins Hsa and CshA/CshB (see Fig. 2). Heterologous expression systems have previously been employed to assess the functions of streptococcal AgI/II proteins in the absence of other streptococcal adhesins (Holmes *et al.*, 1998; Jakubovics *et al.*, 2005b). To investigate the potential of *S. gordonii* AgI/II adhesins SspA and SspB to bind FN, we expressed these proteins independently on the cell surface of *L. lactis*. Adhesion of *L. lactis* expressing SspA or SspB to FN was significantly (*P*<0.05) increased compared with the wild-type parent strain *L. lactis* MG1363 (Fig. 4), confirming that these proteins bind to FN. Sialidase treatment of FN did not significantly affect adherence levels, suggesting that the SspA and SspB proteins do not recognize terminal sialic acid moieties. Although SspA and SspB polypeptides interact with FN, they do not seem to be the major cell-surface adhesins mediating adherence to FN.

## DISCUSSION

The above data demonstrate that *S. gordonii* utilizes several different cell-surface adhesins for recognition of human plasma FN. The mechanism of FN binding by *S. gordonii* has clear differences from the well-characterized interactions between *S. pyogenes* or *Sta. aureus* and FN. For example, soluble FN inhibits adherence of *S. pyogenes* to immobilized FN, whereas soluble-phase FN enhances binding of *S. gordonii* to immobilized host substrates, including gelatin, FN and collagen (Lowrance *et al.*, 1988). The major interactions between *S. pyogenes* or *Sta. aureus* and FN involve recognition of the FN peptide backbone by bacterial cell-surface adhesins (Schwarz-Linek *et al.*, 2003). We found that adhesion of *S. pyogenes* A40 or *Sta. aureus* Newman to FN was not affected by pre-treating FN with sialidase (neuraminidase; data not shown). By contrast, binding of *S. gordonii* was reduced >50 % following desialylation of the FN (Fig. 2a[Fig f2]). Thus, *S. gordonii* recognizes and binds to both the peptide backbone and the carbohydrate decorations on FN.

The genetic basis of FN adhesion by *S. gordonii* has been the subject of several investigations. There is strong evidence that CshA/CshB fibrils on the streptococcal cell surface mediate FN binding. Inactivation of the genes encoding CshA and CshB reduces the ability of *S. gordonii* to bind immobilized FN by approximately 30–40 % (Fig. 1; McNab *et al.*, 1996). Antibodies against the N-terminal region of CshA polypeptide inhibit binding of *S. gordonii* to FN (McNab *et al.*, 1996). Moreover, heterologous expression of CshA on the cell surface of *E. faecalis* promoted adhesion of cells to FN (McNab *et al.*, 1999). More recently, Giomarelli *et al.* (2006) demonstrated that *S. gordonii* mutants lacking Hsa or the AgI/II family proteins SspA and SspB were impaired in their abilities to bind FN. Similarly, we have confirmed that an *hsa* knockout mutant of *S. gordonii* DL1 (Challis) has a significantly reduced capacity to bind FN (Fig. 1). However, in contrast to Giomarelli *et al.* (2006), we were unable to find any reduction in FN binding in a mutant lacking SspA and SspB. Single mutants lacking either SspA or SspB alone were also unimpaired in adhesion to FN (data not shown). It is possible that our laboratory stock of *S. gordonii* DL1 (Challis) has minor differences in cell-surface composition from the variant used by Giomarelli *et al.* (2006). Nevertheless, we found some evidence that, in the absence of FN adhesins CshA/CshB and/or Hsa, FN binding was promoted by SspA and SspB (Fig. 2a[Fig f2] and Fig. 4). We propose that AgI/II proteins play a secondary role in FN binding by *S. gordonii*. FN binding by AgI/II proteins may be of greater importance for streptococci that produce a more restricted range of cell-surface adhesins. Thus, disruption of the genes encoding AgI/II proteins from *Streptococcus intermedius* or *Streptococcus mutans* reduces binding to FN by approximately 75 or 20 %, respectively (Petersen *et al.*, 2002).

**Fig. 5. f5:**
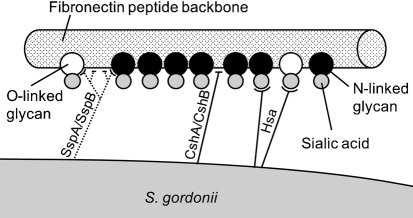
Model to describe the adhesion interactions between *S. gordonii* DL1 and FN. FN is represented as a polypeptide chain (cylinder) with attached O-linked (white circles) or N-linked (black circles) glycans (based on data from Tajiri *et al.*, 2005). Glycans may contain terminal sialic acid residues (grey circles) that are recognized by *S. gordonii* Hsa. *S. gordonii* fibrillar adhesins CshA and CshB also interact with FN by recognizing specific peptide structures in the FN backbone. Secondary interactions occur between *S. gordonii* SspA and SspB and FN that are difficult to observe in the presence of Hsa or CshA/CshB. SspA and SspB adhesins may recognize peptide structures or carbohydrate moieties attached to the FN backbone.

Based on the data presented here and in previous reports, we propose a model for FN binding by *S. gordonii* in which CshA/CshB and Hsa polypeptides are primary adhesins and AgI/II proteins SspA/SspB are secondary components of the interaction. This model is illustrated in Fig. 5. Altogether, nine glycosylation sites have been described in human plasma FN, seven of which are N-linked and two are O-linked (Tajiri *et al.*, 2005). Both N-linked and O-linked carbohydrates contain terminal sialic acid residues, and these are predicted to be the sites of interaction with *S. gordonii* Hsa. Similarly, Hsa interacts with carbohydrate structures in N-linked and O-linked glycans of the platelet receptor GPIb*α* (Takamatsu *et al.*, 2005). Hsa accounted for the total interaction of *S. gordonii* with sialic acids; there was no indication that other adhesins bound sialic acids on either FN or fetuin. It is postulated that CshA and CshB polypeptides bind the peptide backbone of FN. Adhesion by SspA and SspB may also involve recognition of FN peptide structures. However, the crystal structure of a homologous AgI/II protein from *S. mutans* contains a carbohydrate-binding fold (Troffer-Charlier *et al.*, 2002), and it is possible, therefore, that SspA and SspB bind to sialidase-insensitive carbohydrate decorations on FN.

Interactions of Hsa with substrates are dependent upon post-translational processing and targeting to the cell surface. Secretion of Hsa requires the secondary secretion system encoded by *secA2* and *secY2* (Bensing & Sullam, 2002; Bensing & Sullam, 2009). *S. gordonii* Challis has been stored in the laboratory for over 70 years, and at least one clonal line, *S. gordonii* CH1, has acquired a point mutation in the *secA2* gene that prevents the proper secretion of Hsa (Bensing *et al.*, 2004). In contrast to *S. gordonii* DL1 (Challis), no binding of *S. gordonii* CH1 (Challis) to immobilized FN was observed (data not shown). To assess the role of *secA2*, a knockout mutant was constructed in which the *secA2* gene of *S. gordonii* DL1 (Challis) was disrupted by allelic exchange mutagenesis. This strain did not bind FN to any appreciable level (data not shown). Unfortunately, despite several attempts, and despite previous reports of *secA2* complementation in the literature (Bensing *et al.*, 2004), we were unable to produce a construct for functional SecA2 protein expression. Therefore, at present it cannot be confirmed that the *secA2* mutation in *S. gordonii* CH1 (Challis) is the sole cause of the inability of this strain to bind FN. It is possible that co-ordinated *hsa* and *secA2* expression may be essential for the optimal function of the Hsa adhesin. This would explain why expression of *hsa* from a plasmid in *S. gordonii* UB1545(pMSP-*hsa*) did not fully complement the defective adhesion of the *hsa* mutant *S. gordonii* UB1545 to FN (Fig. 3c[Fig f3]).

In summary, the data presented here reveal a mechanism of FN binding in *S. gordonii* that involves multiple cell-surface adhesins Hsa, CshA, CshB, SspA and SspB. We demonstrate that bacterial adhesion to FN is dependent upon recognition of the carbohydrate decorations attached to the FN backbone. This observation calls into question other reports on FN binding by bacteria, where it has often been assumed that bacterial adhesins recognize the FN peptide backbone. Carbohydrate structures on FN may, for example, form receptors for adhesins such as *S. pyogenes* GAPDH, Fbp54 or Shr that do not possess *β*-zipper-forming domains. Interactions between *S. gordonii* and FN carbohydrates are primarily mediated by the sialic-acid-binding glycoprotein Hsa, orthologues of which are expressed by strains of *S. gordonii* and *S. sanguinis* (Plummer & Douglas, 2006; Takamatsu *et al.*, 2006). Genes encoding homologues of CshA, CshB, SspA and SspB are present in the genome sequence of *S. sanguinis* SK36 (Xu *et al.*, 2007), and AgI/II proteins (SspA/SspB homologues) are found in strains of *S. mutans*, *S. oralis*, *S. sobrinus* and *S. intermedius*. Thus it would be expected that combinations of these functions might confer abilities to bind FN by these other organisms. Understanding these FN interactions better may lead to new molecules that would counteract streptococcal infective endocarditis.

## Abbreviations

- FN, fibronectin
- sWGA, succinylated wheatgerm agglutinin
